# Relationship of SARS-CoV-2 Antigen and Reverse Transcription PCR Positivity for Viral Cultures

**DOI:** 10.3201/eid2803.211747

**Published:** 2022-03

**Authors:** Dustin W. Currie, Melisa M. Shah, Phillip P. Salvatore, Laura Ford, Melissa J. Whaley, Jennifer Meece, Lynn Ivacic, Natalie J. Thornburg, Azaibi Tamin, Jennifer L. Harcourt, Jennifer Folster, Magdalena Medrzycki, Shilpi Jain, Phili Wong, Kimberly Goffard, Douglas Gieryn, Juliana Kahrs, Kimberly Langolf, Tara Zochert, Christopher H. Hsu, Hannah L. Kirking, Jacqueline E. Tate, for the CDC COVID-19 Response Epidemiology Field Studies Team

**Affiliations:** Centers for Disease Control and Prevention, Atlanta, Georgia, USA (D.W. Currie, M.M. Shah, P.P. Salvatore, L. Ford, M.J. Whaley, N.J. Thornburg, A. Tamin, J.L. Harcourt, J. Folster, M. Medrzycki, S. Jain, P. Wong, C.H. Hsu, H.L. Kirking, J.E. Tate);; Marshfield Clinic Research Institute, Marshfield, Wisconsin, USA (J. Meece, L. Ivacic);; Winnebago County Health Department, Oshkosh, Wisconsin, USA (K. Goffard, D. Gieryn);; University of Wisconsin‒Oshkosh, Oshkosh (J. Kahrs, K. Langolf, T. Zochert)

**Keywords:** COVID-19, severe acute respiratory syndrome coronavirus 2, SARS-CoV-2, coronaviruses, viruses, coronavirus disease, rapid antigen test, reverse transcription PCR, cycle threshold, respiratory infections, viral cultures, zoonoses

## Abstract

We assessed the relationship between antigen and reverse transcription PCR (RT-PCR) test positivity and successful virus isolation. We found that antigen test results were more predictive of virus recovery than RT-PCR results. However, virus was isolated from some antigen-negative and RT-PCR‒positive paired specimens, providing support for the Centers for Disease Control and Prevention antigen testing algorithm.

Antigen platforms for severe acute respiratory syndrome coronavirus 2 (SARS-CoV-2) diagnostic testing have rapid turnaround time, are easy to use, and are less expensive than real-time reverse transcription PCR (RT-PCR) diagnostic testing. Using RT-PCR as the reference test, performance evaluations of the Abbott BinaxNOW COVID-19 Antigen Card Test (https://www.abbott.com) reported a high specificity (>98%) (*1*–*4*) but lower sensitivity, ranging from 64.2% to 89.0% for symptomatic persons (*2*–*4*) and 35.8% to 70.2% for asymptomatic persons (*2*,*3*). However, other studies have demonstrated a period of prolonged positivity for RT-PCR testing beyond which virus has been isolated (*5*,*6*). Therefore, a comprehensive examination of antigen test performance characteristics in identifying infectious persons who have SARS-CoV-2 infections requires comparison with multiple data points, including RT-PCR test positivity and the ability to isolate the virus (a marker for infectiousness) (*5*–*7*). In this study, we expand on a previous report (*1*) that examined performance of antigen testing relative to RT-PCR by reporting virus isolation data for persons who had positive results by antigen test or RT-PCR.

## The Study

The study population and testing methods have been described (*1*). Persons were recruited at a free, appointment-based, community antigen testing site in Winnebago County, Wisconsin, USA. Approximately 30 minutes after providing an initial nasal swab specimen for antigen testing, 2 additional self-collected specimens were collected under Centers of Disease Control and Prevention (CDC) staff supervision from the anterior nares simultaneously in an alternating fashion to maximize uniformity.

Of 2 simultaneous swab specimens, we used 1 specimen for rapid antigen testing by the Abbott BinaxNOW SARS-CoV-2 Antigen Card Test, a point-of-care lateral flow test with results available within 15 minutes of specimen collection. We placed the other specimen in viral transport medium and transported it on ice to the Marshfield Clinical Research Institute laboratory (Marshfield, Wisconsin, USA) for RT-PCR testing. Specimens with a cycle threshold (C_t_) value <37 for at least 2 of 3 SARS-CoV-2 gene targets (open reading frame 1ab, spike gene, and nucleocapsid gene) were considered positive, according to the instructions of the manufacturer (TaqPath COVID-19 Combo Kit; Thermo Fisher Scientific, https://www.thermofisher.com).

We attempted viral culture at a CDC laboratory for all participants testing positive by RT-PCR or antigen test by using Vero-CCL81 cells, which were inoculated with clinical specimens, and observed daily for 7 days (*8*). All cultures that had a visible cytopathic effect were used for RNA extraction and SARS-CoV-2 RT-PCR confirmation. Any specimen that showed a cytopathic effect, was positive by RT-PCR, and had a C_t_ >2 lower than that for the original clinical specimen was considered culture positive.

We collected symptoms at time of specimen collection, symptom onset date, and exposure history by using paper questionnaires and entered data into REDCap database version 11.0.3 (https://www.vumc.org/dbmi/redcap). Participants reporting >1 of 15 symptoms at the time of specimen collection were considered symptomatic. Possible symptoms were fever, rigors, nasal congestion, sore throat, shortness of breath, headache, diarrhea, loss of taste, loss of smell, chills, muscle aches, fatigue, cough, nausea, and abdominal pain.

We define known exposure as being within 6 feet of a person who tested positive for SARS-CoV-2 within the last 14 days for >15 minutes over a 24-hour period. We analyzed data by using SAS version 9.4 (https://www.sas.com). We made comparisons by using Kruskal‒Wallis test for continuous variables or χ^2^ tests for categorical variables; statistical significance was defined as α<0.05. This analysis was reviewed by CDC and was conducted consistent with applicable federal law and CDC policy.

During November 16–December 15, 2020, we collected 2,112 specimen pairs that had valid results for PCR and antigen tests; most (56.3%) participants were symptomatic (age range 5–95 years, median 42 years). Of 2,112 specimen pairs, 334 (15.8%) were positive by RT-PCR, 269 (12.7%) were positive by antigen test, and 200 (9.5%) had recoverable virus (culture positive). Of the 200 culture positive specimen pairs that had a positive RT-PCR result, 191 (95.5%) had a positive antigen test result. Positive predictive value (PPV) of antigen test for culture positivity (191/269, 71.0%) (Table 1) was higher than PPV for RT-PCR (200/334, 59.9%). Virus was successfully isolated from 191 (71.5%) of 267 specimen pairs with concordant positive antigen/RT-PCR results, 9 (13.4%) of 67 specimen pairs with positive RT-PCR and negative antigen test results, and 0 of 2 specimen pairs with positive antigen and negative RT-PCR test results.

**Table 1:** Positive predictive value of the BinaxNOW COVID-19 Antigen Card Test and RT-PCR relative to viral culture, Winnebago County, Wisconsin, USA, November‒December 2020*

SARS-CoV-2 diagnostic test result	No. culture positive	No. culture negative	Total	Positive predictive value, %
BinaxNOW positive	191	78	269	71.0
RT-PCR positive	200	134	334	59.9

*BinaxNOW, https://www.abbott.com. RT-PCR, reverse transcription PCR; SARS-CoV-2, severe acute respiratory syndrome coronavirus 2.

All participants who had culture-positive specimens and false-negative antigen tests were symptomatic (7/9; 77.8%) or had a known exposure in the past 14 days (5/9; 55.6%). Among culture-positive symptomatic participants, those who had false-negative antigen and concordant positive antigen/RT-PCR results were tested a similar number of days after symptom onset (median 2 days vs. 3 days) (Table 2). The 2 persons who had recoverable virus and false-negative antigen test results and who were asymptomatic at the time of testing had known exposures the day before testing. For those who had recoverable virus, nucleocapsid gene C_t_ values were significantly lower in those with concordant positive results (median 19.1, interquartile range 17.1–21.3) than those who had false-negative antigen test results (median 26.6, interquartile range 25.6–31.0) (p<0.0001) (Table 2; Figure).

**Table 2:** Symptoms and exposure history of persons testing positive for SARS-CoV-2, stratified by ability to culture virus and RT-PCR/antigen test concordance, Winnebago County, Wisconsin, USA, November-December 2020*

Characteristic	Culture positive				Culture negative		
	RT-PCR+/ antigen–, n = 9	RT-PCR+/ antigen+, n = 191	All, n = 200		RT-PCR+/ antigen–, n = 58	RT-PCR+/ antigen+, n = 76	All, n = 134†
Symptomatic							
Current symptoms	7 (77.8)	165 (88.2)	172 (87.8)		43 (73.7)	67 (88.2)	109 (82.0)
No current symptoms	2 (22.2)	22 (11.8)	24 (12.2)		15 (26.3)	9 (11.8)	24 (18.0)
Unknown/missing	0	4	4		1		1
Meets CSTE clinical criteria‡							
Yes	7 (77.8)	142 (74.3)	149 (74.5)		36 (62.1)	60 (78.9)	96 (71.6)
No	2 (22.2)	49 (25.7)	51 (25.5)		22 (37.9)	16 (21.1)	38 (28.4)
Days from symptom onset to specimen collection, median (IQR)	2 (1–6 d)	3 (1–4)	3 (1–5)		3 (1–10)	4 (2–7)	4 (2–7)
Known exposure in previous 14 d							
Yes	5 (55.6)	111 (58.7)	116 (58.6)		34 (59.7	39 (52.0)	73 (55.3)
No	1 (11.1)	42 (22.2)	43 (21.7)		19 (33.3)	22 (29.3)	41 (31.1)
Unknown	3 (33.3)	36 (19.1)	39 (19.7)		4 (7.0)	14 (18.7)	18 (13.6
Missing	0	2	2		1	1	2
Days since last known exposure, median (IQR)	2 (0.5–4)	4 (0–6)	4 (0–6)		2 (0–4 d)	3 (0–7)	2 (0–6)
N gene C_t_ value, median (IQR)	26.6 (25.6–31.0)	19.1 (17.1–21.3)	19.2 (17.2–21.7)		30.9 (29.3–33.4)	24.3 (21.1–27.7)	27.9 (23.8–30.9)

*Values are no. (%) except as indicated. CSTE, Council of State and Territorial Epidemiologists; C_t ,_ cycle threshold; IQR, interquartile range; N, nucleocapsid; RT-PCR, reverse transcription PCR; SARS-CoV-2, severe acute respiratory syndrome coronavirus 2; +, positive; ‒, negative. †All specimen pairs included tested positive by RT-PCR; the 2 participants who had false-positive antigen test results, both of whom were culture negative, were excluded. ‡CSTE clinical criteria are met if the case-patient has either cough, shortness of breath, loss of taste, or loss of smell, or >2 of the following symptoms: fever, chills, myalgia, headache, or sore throat.

**Figure:**
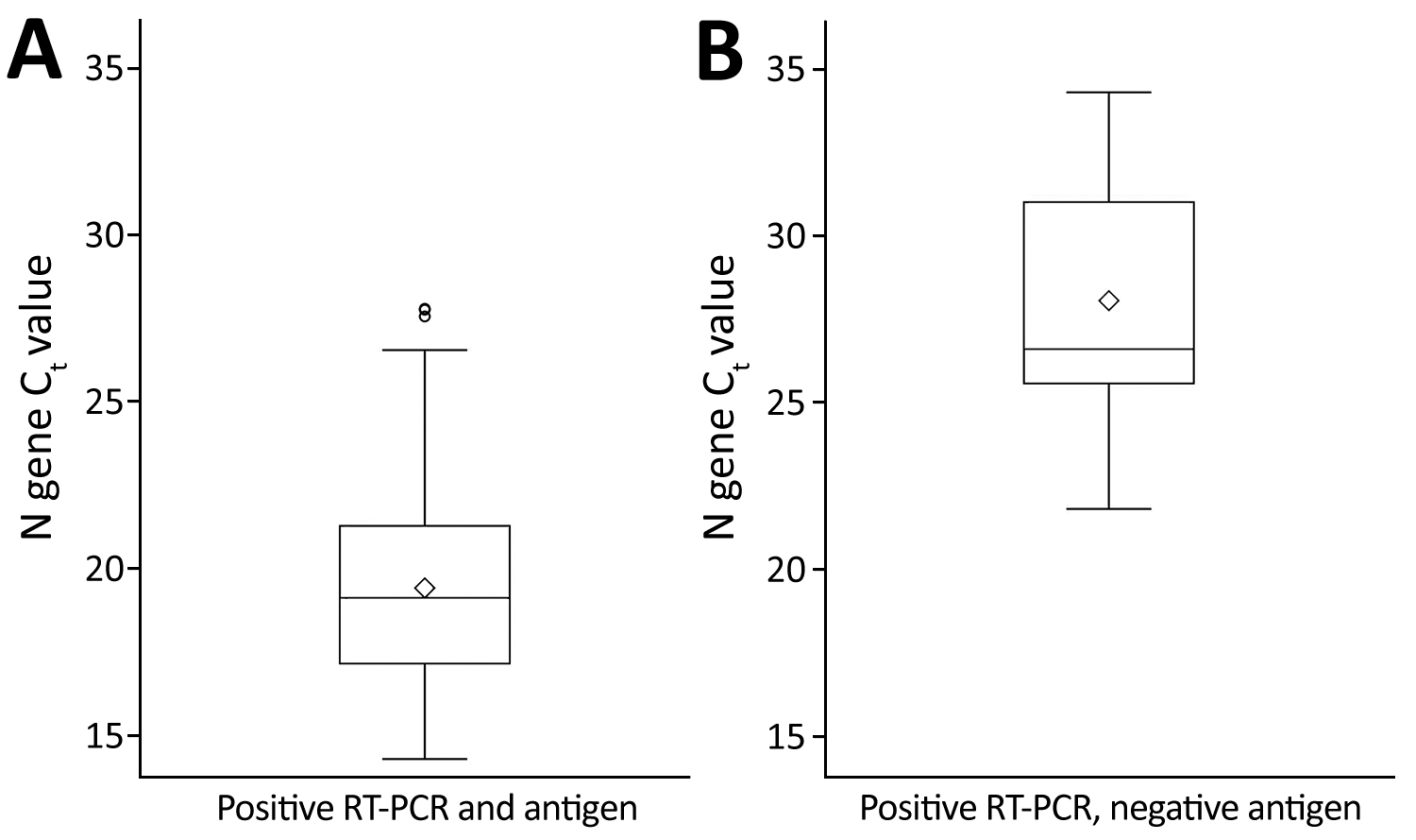
Box plots of C_t_ values among participants with recoverable virus who had concordant positive SARS-CoV-2 RT-PCR and antigen test results (A) compared with those who had positive RT-PCR and negative antigen test results (B), Winnebago County, Wisconsin, USA, November–December 2020. The difference between the 2 groups was significant (p<0.0001). Diamonds indicate means, boxes indicate the first quartile through the third quartile, horizontal bars in boxes indicate medians, and errors bars indicate minimum values to maximum values; outliers are plotted as individual circles. C_t_, cycle threshold; RT-PCR, reverse transcription PCR; SARS-CoV-2, severe acute respiratory syndrome coronavirus 2.

## Conclusions

Consistent with previous studies assessing the relationship between antigen tests, RT-PCR, and ability to culture virus (*9*–*11*), we found that SARS-CoV-2 was more likely to be recovered among specimen pairs for which antigen test and RT-PCR results were positive than among pairs in which antigen test results were negative and RT-PCR results were positive. Although some studies have shown similar PPV for viral culture when comparing RT-PCR and antigen tests (*12*), we found higher PPV for the antigen test than for RT-PCR (*13*), suggesting that antigen test positivity might be a better marker of infectiousness than a positive RT-PCR result. However, a small but nontrivial proportion of samples that had negative antigen and positive RT-PCR results had recoverable virus, suggesting that antigen tests are misclassifying some infectious persons as SARS-CoV-2 negative. This finding, consistent with those of similar studies (*6*–*11*), suggests that lower sensitivity of antigen tests when compared with RT-PCR cannot be attributed exclusively to lingering positive RT-PCR results for persons who are no longer infectious.

Symptoms on the day of testing for most infectious persons who had false-negative antigen test results suggests that CDC’s current antigen testing guidance, which recommends confirmatory RT-PCR testing after negative antigen test results for symptomatic persons in community settings (*14*), is appropriate. Both asymptomatic participants who had false-negative antigen test results and recoverable virus had exposures within the previous 48 hours. Therefore, all participants who had false-negative antigen test results were unlikely to infect others if following CDC guidance because they would have been advised to quarantine because of exposure (asymptomatic close contacts) or while awaiting confirmatory RT-PCR results (symptomatic persons).

One limitation of this study was that although recoverable virus is indicative of infectiousness, lack of ability to isolate virus does not necessarily imply lack of infectiousness (*15*). Symptom status was only measured at the time of testing. Because we did not attempt virus isolation on antigen-negative and RT-PCR‒negative specimens, PPV was the only reported measure of agreement between antigen test, RT-PCR, and recoverable virus in culture. Because RT-PCR testing was not performed with calibrators, we are not able to report values in copies/milliliter. Finally, this investigation assessed only the BinaxNOW antigen testing platform.

This study suggests that antigen test positivity is more predictive of infectiousness than RT-PCR test positivity. However, false-negative antigen test results can be obtained for infectious persons, especially among those with symptoms, supporting CDC recommendations to follow negative antigen testing among symptomatic persons with RT-PCR confirmatory testing within 48 hours (*14*).

## References

[1] Shah MM, Salvatore PP, Ford L, Kamitani E, Whaley MJ, Mitchell K, et al. Performance of repeat BinaxNOW SARS-CoV-2 antigen testing in a community setting, Wisconsin, November‒December 2020. Clin Infect Dis. 2021;73(Suppl 1):S54–7. 10.1093/cid/ciab309 33909068 PMC8135465

[2] Prince-Guerra JL, Almendares O, Nolen LD, Gunn JKL, Dale AP, Buono SA, et al. Evaluation of Abbott BinaxNOW rapid antigen test for SARS-CoV-2 infection at two community-based testing sites—Pima County, Arizona, November 3–17, 2020. MMWR Morb Mortal Wkly Rep. 2021;70:100–5. 10.15585/mmwr.mm7003e3 33476316 PMC7821766

[3] Pollock NR, Jacobs JR, Tran K, Cranston AE, Smith S, O’Kane CY, et al. Performance and implementation evaluation of the Abbott BinaxNOW rapid antigen test in a high throughput drive-through community testing site in Massachusetts. J Clin Microbiol. 2021;59:e00083–21. 10.1128/JCM.00083-21 33622768 PMC8091851

[4] Pilarowski G, Marquez C, Rubio L, Peng J, Martinez J, Black D, et al. Field performance and public health response using the BinaxNOW^TM^ rapid SARS-CoV-2 antigen detection assay during community-based testing. Clin Infect Dis. 2021;73:e3098–1. 10.1093/cid/ciaa1890 33367619 PMC7799223

[5] Jefferson T, Spencer EA, Brassey J, Heneghan C. Viral cultures for COVID-19 infectious potential assessment: a systematic review. Clin Infect Dis. 2020;•••: Epub ahead of print. 33270107 10.1093/cid/ciaa1764 PMC7799320

[6] Doshi P, Powers JH. Determining the infectious potential of individuals with positive reverse transcription polymerase chain reaction severe acute respiratory syndrome 2 tests. Clin Infect Dis. 2020;•••: Epub ahead of print. 33277652 10.1093/cid/ciaa1819 PMC7799214

[7] Steinlin-Schopfer J, Barbani MT, Kamgang R, Zwahlen M, Suter-Riniker F, Dijkman R. Evaluation of the Roche antigen rapid test and a cell culture-based assay compared to rRT-PCR for the detection of SARS-CoV-2: a contribution to the discussion about SARS-CoV-2 diagnostic tests and contagiousness. J Clin Virol Plus. 2021;1:100020. 10.1016/j.jcvp.2021.100020 35262007 PMC8106823

[8] Harcourt J, Tamin A, Lu X, Kamili S, Sakthivel SK, Murray J, et al. Severe acute respiratory syndrome coronavirus 2 from patient with coronavirus disease, United States. Emerg Infect Dis. 2020;26:1266–73. 10.3201/eid2606.200516 32160149 PMC7258473

[9] Strömer A, Rose R, Schäfer M, Schön F, Vollersen A, Lorentz T, et al. Performance of a point-of-care test for the rapid detection of SARS-CoV-2 antigen. Microorganisms. 2020;9:58. 10.3390/microorganisms9010058 33379279 PMC7823488

[10] Kohmer N, Toptan T, Pallas C, Karaca O, Pfeiffer A, Westhaus S, et al. The comparative clinical performance of four SARS-CoV-2 rapid antigen tests and their correlation to infectivity in vitro. J Clin Med. 2021;10:328. 10.3390/jcm10020328 33477365 PMC7830733

[11] McKay SL, Tobolowsky FA, Moritz ED, Hatfield KM, Bhatnagar A, LaVoie SP, et al.; CDC Infection Prevention and Control Team and the CDC COVID-19 Surge Laboratory Group. Performance evaluation of serial SARS-CoV-2 rapid antigen testing during a nursing home outbreak. Ann Intern Med. 2021;174:945–51. 10.7326/M21-0422 33900791 PMC8108910

[12] Ford L, Lee C, Pray IW, Cole D, Bigouette JP, Abedi GR, et al. Epidemiologic characteristics associated with SARS-CoV-2 antigen-based test results, rRT-PCR cycle threshold values, subgenomic RNA, and viral culture results from university testing. Clin Infect Dis. 2021;73:e1345–55. 10.1093/cid/ciab303 33846714 PMC8083323

[13] Pekosz A, Parvu V, Li M, Andrews JC, Manabe YC, Kodsi S, et al. Antigen-based testing but not real-time polymerase chain reaction correlates with severe acute respiratory syndrome coronavirus 2 viral culture. Clin Infect Dis. 2021;73:e2861–6. 10.1093/cid/ciaa1706 33479756 PMC7929138

[14] Centers for Disease Control and Prevention. Using antigen tests for SARS-CoV-2 in community settings, 2021 [cited 201 Jun 5]. https://www.cdc.gov/coronavirus/2019-ncov/lab/resources/antigen-tests-guidelines.html#using-antigen-tests-community-settings

[15] Gniazdowski V, Morris CP, Wohl S, Mehoke T, Ramakrishnan S, Thielen P, et al. Repeat COVID-19 molecular testing: correlation of SARS-CoV-2 culture with molecular assays and cycle thresholds. Clin Infect Dis. 2021;73:e860–9. 10.1093/cid/ciaa1616 33104776 PMC7665437

